# Optimization of Plasmid Curing from Genetically Engineered *Clostridium autoethanogenum*

**DOI:** 10.1021/acssynbio.5c00456

**Published:** 2025-12-02

**Authors:** Victoria Chinonyerem Udemezue, Kurshedaktar Majibullah Shaikh, Mariia Vorontsova, Kaspar Valgepea

**Affiliations:** Institute of Bioengineering, 37546 University of Tartu, 50411 Tartu, Estonia

**Keywords:** plasmid curing, CRISPR/Cas9, gas fermentation, acetogen, *Clostridium autoethanogenum*

## Abstract

Accumulation of greenhouse gases from combustion of fossil fuels drives climate change and threatens biosustainability on Earth. Microbial gas fermentation realizes the capture of CO_2_ toward biomanufacturing of value-added products. Acetogens are attractive biocatalysts here, as they use CO_2_ as their sole carbon source with H_2_. Metabolic engineering of novel cell factories is, however, hampered by the slow and complex genetic engineering workflows. Here, we developed different approaches to optimize plasmid curing from genetically engineered strains of the model acetogen *Clostridium autoethanogenum*. Interestingly, a CRISPR/Cas9-based curing plasmid (C-plasmid) targeting the origin of replication both in the target editing plasmid and in the C-plasmid did not improve curing over a non-targeting control plasmid. Strikingly, plasmid curing by making cells electrocompetent (ECCs) and by non-transformative electroporation of ECCs or buffer-washed glycerol stocks showed 14–100% curing efficiencies across the approaches for five different genetically engineered *C. autoethanogenum* strains. The most time-efficient approach with non-transformative electroporation of buffer-washed glycerol stocks also cured an editing plasmid from *Escherichia coli*, with ∼97% efficiency. This work both improves genetic engineering workflows for *C. autoethanogenum* by significantly accelerating plasmid curing and offers methods to potentially ease plasmid curing in other microbes.

## Graphical abstract

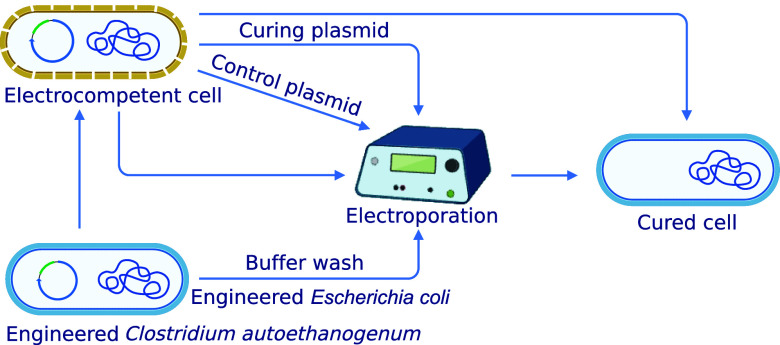

## Introduction

Buildup of greenhouse gases from combustion of fossil fuels by human activities is driving climate change and threatening life on Earth. Microbial gas fermentation has emerged as an attractive biomanufacturing technology for recycling carbon oxides, CO_2_ and CO, from waste gases and gasified solid organic waste into fuels, chemicals, and feed protein in place of fossil fuels.^1^ Acetogens are attractive biocatalysts for this, as they use gases as their sole source of carbon and energy using the most energy-efficient known C1-fixation pathway, the Wood–Ljungdahl pathway.^2,3^ Importantly, acetogens can natively produce and have been engineered to produce a variety of value-added products, e.g., acetate, ethanol, 2,3-butanediol, acetone, and isopropanol.^4^ Acetogen gas fermentation has been deployed industrially by LanzaTech since 2018 for ethanol production using the acetogen *Clostridium autoethanogenum*.^1^

Although the genetic tools for metabolic engineering of acetogens have improved considerably in recent years, workflows are still slow.^4^ For instance, ∼10 to 10^3^ CFU/μgDNA transformation efficiencies for the model-acetogen *C. autoethanogenum* are orders-of-magnitude lower than those for model-microbes, such as *Escherichia coli*. Additionally, recombinant plasmids need a specific methylation pattern to avoid being recognized as foreign and degraded by endogenous restrictases.^5^ Furthermore, *C. autoethanogenum* is an obligate anaerobe that must be handled under strictly anaerobic conditions, which complicates workflows. Lastly, plasmid curing is problematic in *C. autoethanogenum* and other acetogens, which slows work.^6^

Curing of a recombinant plasmid that has realized its targeted genomic modifications is important for reliable phenotypic characterization of the mutant strain without the confounding effects from plasmid expression burden^6^ and for subsequent genetic engineering of the mutant.^7^ Compared to techniques for plasmid transformation, techniques for plasmid removal have been less studied and optimized.^8,9^ The standard approach for plasmid curing is serial strain subculturing in non-selective media.^10^ However, due to the very stable origin of replication and slow growth, this approach is impractical in acetogens. For instance, we have observed great variability in the number of subcultures and time needed for curing different plasmids from various *C. autoethanogenum* strains, ∼340 to ∼1100 h,^10–12^ and in extreme cases, up to 90 subcultures could be needed (J. Dykstra, personal communication).^13^ Alternatively, curing agents like rifampicin, lawsone,^14^ sodium lauryl sulfate, ethidium bromide, acridine orange,^15^ and 5-fluoroorotic acid could be used to inhibit plasmid replication,^8^ but these can induce stress responses, affect gene expression, or cause mutations.^16^ Another option is to use elevated growth temperatures,^8,17^ with a temperature-sensitive origin of replication like *repA*101ts,^18^ but this necessitates handling plasmid-carrying cells at suboptimal temperature, which could lead to slower growth and stress responses.^19^ Recently, a CRISPR/Cas9 plasmid-based curing system that targets both recombinant plasmids to be cured and itself was developed and achieved 80% plasmid curing efficiency in *E. coli* and *Pseudomonas putida*,^9^ while a CRISPR/Cas9 plasmid targeting only the recombinant plasmid showed 83–100% curing efficiencies in *Clostridium saccharoperbutylacetonicum* and*Eubacterium limosum*.^20,21^ Thus, we aimed in this work to develop a similar CRISPR/Cas9-based system for optimizing plasmid curing from the model acetogen *C. autoethanogenum*. While our CRISPR/Cas9-based curing plasmid did not improve curing over a non-targeting control plasmid, we surprisingly discovered three other curing approaches not involving CRISPR/Cas9-plasmids showing 14–100% plasmid curing efficiencies.

## Results and Discussion

### CRISPR/Cas9-Based Curing Plasmid Construction

The CRISPR/nCas9 editing plasmids (E-plasmid) we use for making genetic modifications in *C. autoethanogenum* include the nCas9 gene for nicking DNA directed by a gRNA, homology arms for genomic repair, the *mlsR* resistance gene for plasmid selection using clarithromycin (CLA), and the *repH* (from plasmid pCB102) origin of replication. To create a CRISPR/Cas9-based curing plasmid (C-plasmid) that would target the E-plasmid and itself, we replaced E-plasmid components with the *repA* (from pBP1) origin of replication to allow both plasmids to coexist,^22^ the *catP* resistance gene for C-plasmid selection using thiamphenicol (TMP), and the Cas9 for cutting target DNA (Figure S1). We directed Cas9 to cut both the E-plasmid and itself using one gRNA targeting the ColE1-like origin of replication^9^ on both E- and C-plasmids (no other region was targeted). We also constructed a variant of the C-plasmid with a non-targeting gRNA to serve as a control (N-plasmid).

During gRNA swapping for the C-plasmid, two of the three designed gRNAs (ColE1_gRNA2 and ColE1_gRNA3) yielded colonies (8–9) from *E. coli* cloning. While PCR products had the expected size (Figure S2A), sequencing showed that the extracted plasmids with the expected size were either the parent plasmid or missed some parts of the gRNA expression cassette, and the other six plasmids with smaller sizes missed genetic parts: gRNA, part of Cas9, or Cas9 promoter (Figures S2B–D and S3A). This suggests that both Cas9 and the ColE1-targeting gRNA might have been transcribed in *E. coli* as plasmid fragment deletion has been observed before during Cas9-based plasmid construction.^23^ Alternatively, *E. coli* transcription machinery may have adapted to express the C-plasmid with the resistance gene, excluding part of the Cas9 machinery to avoid toxicity. The possibility of Cas9 causing the observed plasmid fragmentation was unexpected to us at that point, since we were not aware that the Pthl promoter commonly used in Clostridia could trigger transcription in *E. coli*.^24^ To circumvent this issue, we decided to replace the Pthl promoter with a native promoter of *C. autoethanogenum* (CAETHG_4038 promoter^25^) showing strong expression of downstream genes during both heterotrophic and autotrophic growth. This indeed enabled the successful construction of both the N-plasmid (named pGFT165) and the C-plasmid with the complete gRNA expression cassette (Figure S3B) using the ColE1_gRNA3 (pGFT167).

### Electroporation of *C. autoethanogenum* with a C-Plasmid

To test the functionality of the constructed C-plasmid for plasmid curing (Figure 1A,B), the appropriately methylated^5^ C-plasmid was used for electroporation into two genetically engineered *C. autoethanogenum* LAbrini strains carrying the E-plasmid used to execute deletions of dihydrolipoamide dehydrogenase (Δ*lpdA* strain) or glycine cleavage system H protein and corrinoid activation/regeneration protein (Δ*gcvH*Δ*acsV* strain) genes (strains constructed by fellow group members). We note that curing of the E-plasmid from Δ*lpdA* failed during eight rounds of non-selective subculturing (Table S1). For controls, we electroporated the N-plasmid into (a) Δ*lpdA* to verify that two plasmids carrying either *repH* or *repA* can coexist in cells; and (b) LAbrini (*C. autoethanogenum*parental strain without E-plasmid) to verify that electroporation was executed appropriately.

**Figure 1:**
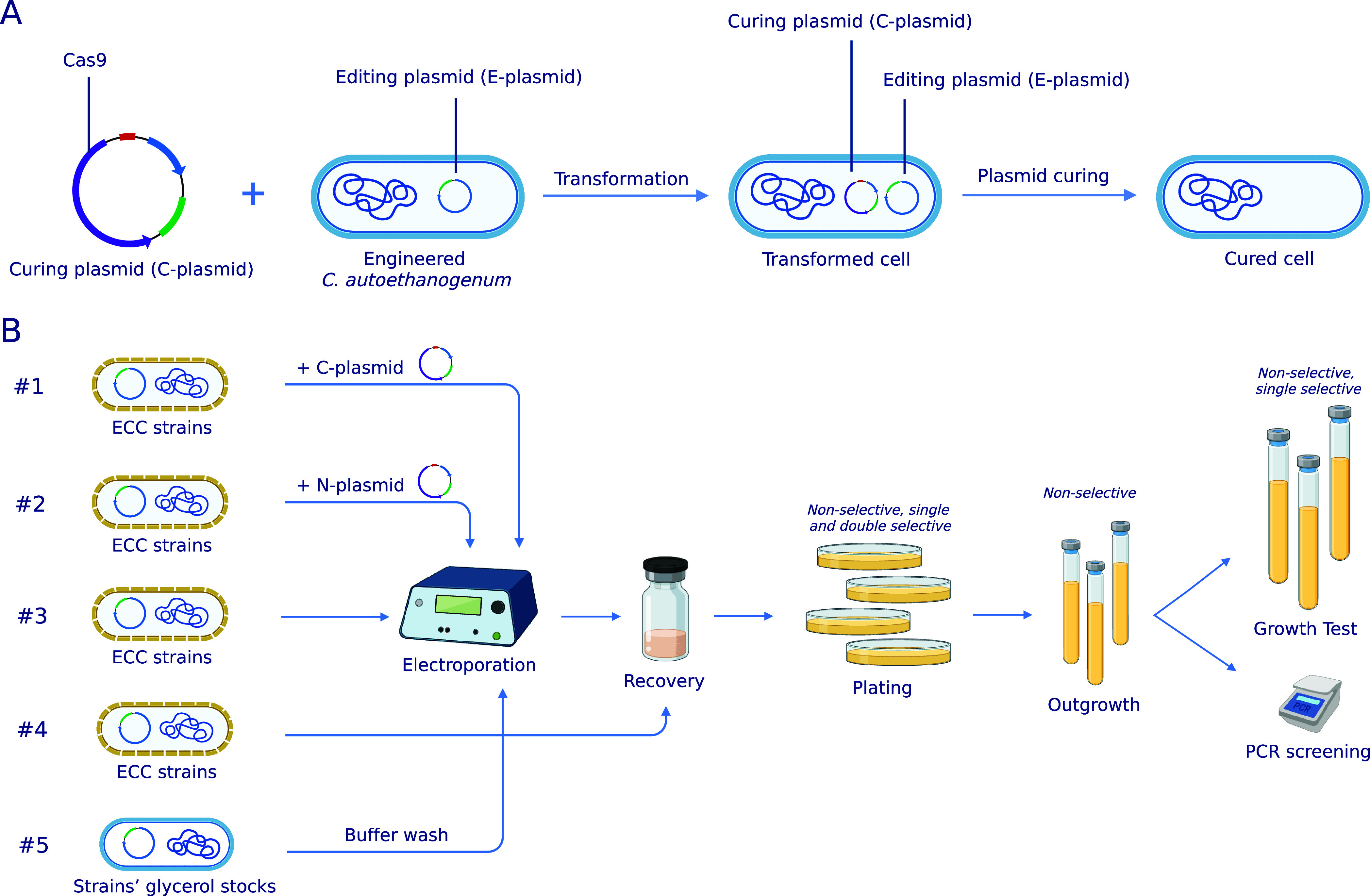
Plasmid curing approaches developed in this work. (A) CRISPR/Cas9-based plasmid curing approach, where the C-plasmid cures both the E-plasmid and itself. (B) All plasmid curing approaches developed in this work. ECC, electrocompetent cells. Created with BioRender.com.

All recovery cultures were first plated on double-selective (TMP + CLA) agar to select for colonies carrying both plasmids (TMP selecting for C/N-plasmid and CLA for E-plasmid); single-selective (TMP or CLA) agar to select for colonies with either plasmid; and nonselective (NS) agar to grow all colonies, including plasmid-cured ones (Table S2). The control electroporation with the N-plasmid and LAbrini yielded colonies on TMP agar, demonstrating successful electroporation of one of the constructed plasmids. This was supported by the second control of the N-plasmid with Δ*lpdA* (carrying E-plasmid) producing colonies on TMP plates, showing that *repH*- and *repA*-bearing plasmids are compatible.^26,27^ Importantly, no colonies for either genetically engineered strain or LAbrini electroporated with the C-plasmid were observed on double-selective or TMP agar, suggesting that the C-plasmid could cure at least itself from all cells (no. 1 on Figure 1B). In fact, a large fraction of cells had lost the E-plasmid since 2-to-5-times more colonies formed on NS vs CLA plates for Δ*lpdA* and Δ*gcvH*Δ*acsV* strains electroporated with the C-plasmid. Surprisingly, the latter was also seen when the deletion strains were electroporated with the N-plasmid while colonies appeared on double-selective agar (no. 2 in Figure 1B). The former could imply that electroporation with the N-plasmid somehow also leads to curing of the E-plasmid. Since this was unexpected, we performed liquid growth tests to verify colony count results (Figure 1B). For this, 10–12 colonies from the NS plates of both deletion strains electroporated with C- or N-plasmid were randomly selected and inoculated into liquid NS YTF, TMP YTF, and CLA YTF media. This growth test confirmed the results from plate counts as cells had lost plasmids with 75–90% efficiency when transformed with either C- or N-plasmid (Figure 2). These efficiencies are similar to the 80–100% curing efficiencies achieved in *E. coli*, *P. putida*, *C. saccharoperbutylacetonicum*, and *E. limosum* using a CRISPR/Cas9 plasmid-based approach.^9,20,21^ We also used PCR and gel-electrophoresis to check for plasmid curing (Table S3) but saw inconsistent results compared to growth tests and plate counts. Detection of plasmid DNA from colonies that cannot grow in selective medium has been seen before.^17^

**Figure 2:**
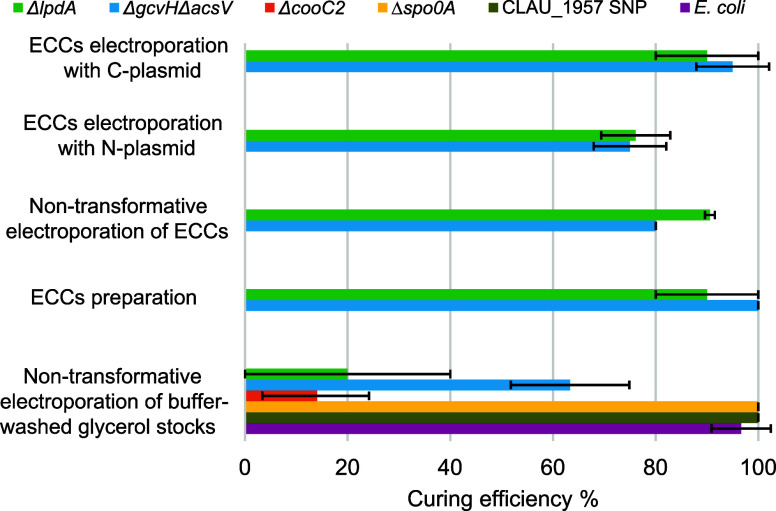
Plasmid curing efficiencies of the approaches developed in this work. Bars represent average and errors bars standard deviation across two-to-four bioreplicates. ECCs, electrocompetent cells.

### Non-transformative Electroporation and Other Approaches Cure Plasmids

Curing of plasmids with the N-plasmid suggests that curing occurred possibly due to other factors than transformation of a new plasmid, e.g., electroporation or electrocompetent cell (ECCs) preparation. Thus, we tested two additional approaches for plasmid curing from both deletion strains: non-transformative (without plasmid) electroporation of ECCs (no. 3 on Figure 1B); and making cells electrocompetent, i.e., ECCs (no. 4 on Figure 1B). Remarkably, both led to plasmid curing (Figure 2). Non-transformative electroporation of ECCs yielded 80–90% curing efficiencies, indicating that transformation of a new plasmid is not needed for curing existing ones. What is more, even electroporation is not essential for plasmid curing as preparation of ECCs for the deletion strains cured plasmids with 90–100% efficiency.

We next attempted to further optimize curing by testing the effect of omitting the first three time-consuming steps of ECCs preparation before electroporation to speed up the curing approach, as this would be faster than curing by preparing ECCs with the complete protocol (no. 4 on Figure 1B). Thus, for this faster approach, glycerol stocks of deletion strains were thawed and washed twice with SMP buffer, to remove salts for electroporation, before non-transformative electroporation (no. 5 in Figure 1B). Notably, this approach also led to plasmid curing with 20–63% efficiencies (Figure 2). Despite these values being lower (*p*-value < 0.05) compared to those of approach no. 1 (Table S4), approach no. 5 is the most time-efficient in our experience for curing plasmids from *C. autoethanogenum*.

We then validated the functionality of this fastest approach with three more genetically engineered *C. autoethanogenum* strains: (1) LAbrini with a deletion of cobyrinic acid ac-diamide synthase gene (Δ*cooC2*; strain constructed by a fellow group member); (2) JA1–1 (“wild-type”) with a deletion of sporulation transcriptional activator Spo0A (Δ*spo0A*; constructed previously^11^); and (3) JA1–1 carrying a single nucleotide polymorphism (SNP) in a two-component transcriptional regulator (CLAU_1957 SNP; constructed previously^11^). Curing efficiencies of 14–100% were observed (Figure 2). Although the overall curing efficiency of the fastest approach no. 5 is lower in LAbrini strains compared to the other approaches we developed (Figure 2), it is reasonably high to obtain cured colonies from first screening, and importantly, it is ∼102 h faster than other methods that usually take ∼386 h, since it lacks the first three steps of ECCs preparation. Also, the fastest developed approach could speed up plasmid curing from *C. autoethanogenum* by up to a month as it takes ∼284 h compared to the standard approach of serial nonselective subculturing that can take up to ∼1100 h^10–12^ or be unsuccessful altogether (Table S1). Interestingly, our fastest approach for curing E-plasmids (no. 5) did not affect maintenance of the *C. autoethanogenum* native plasmid pCA^30^ as it was retained in all three genetically engineered LAbrini strains cured of E-plasmids (Figure S4). Lastly, we tested if approach no. 5 could cure plasmids in another microbe. Indeed, an E-plasmid carrying both *repH* and ColE1-like origin of replication was cured with ∼97% efficiency from *E. coli* NEBExpress by 10% glycerol wash of the glycerol stock and non-transformative electroporation (Figure 2).

In conclusion, we tested the functionality of a CRISPR/Cas9-based approach and developed other approaches for curing *repH*- and ColE1-bearing plasmids from *C. autoethanogenum* and *E. coli*. Curing efficiencies of 14–100% from five genetically engineered *C. autoethanogenum* strains and *E. coli* were observed across the approaches, showing that multiple approaches can be more efficient than the standard approach of serial non-selective subculturing. This work both improves genetic engineering workflows for *C. autoethanogenum* by significantly accelerating plasmid curing and offers methods to potentially ease plasmid curing in other microbes.

## Methods

### Strains and Growth Media

*E. coli* strains, NEB Turbo and NEBExpress (New England Biolabs), were used for cloning, plasmid construction, and propagation. The latter strain was also used for the propagation of plasmids prior to electroporation to achieve a methylation pattern compatible with *C. autoethanogenum*.^5^ *E. coli* strains were routinely cultivated in Lysogeny Broth (LB) medium supplemented with ampicillin (100 μg/mL) aerobically at 37 °C. The *C. autoethanogenum* strain LAbrini^11^ (DSM 115981) was used as a control for electroporation. Three genetically engineered LAbrini strains with gene deletions (Δ*lpdA*, Δ*gcvH*Δ*acsV*, and Δ*cooC2*) constructed by fellow group members using published methodology^10,11^ and two genetically engineered *C. autoethanogenum* JA1–1 strains (Δ*spo0A* and CLAU_1957 SNP) constructed previously^11^ harboring respective E-plasmids were used to test our plasmid curing approaches for *C. autoethanogenum*. *E. coli* NEBExpress transformed with the E-plasmid previously used for *spo0A* deletion in JA1–1 (pGFT107)^11^ was used to test the plasmid curing approach no. 5 for *E. coli*. *C. autoethanogenum* strains were handled anaerobically and grown in YTF medium^10^ or YTF supplemented with CLA (4 μg/mL for agar and 6 μg/mL for liquid), TMP (15 μg/mL), or both. Plasmid and genomic DNA extractions were done as previously reported.^10^ Table S5 lists the strains used in this study.

### Plasmid Construction

Plasmid pGFT096, previously constructed by fellow group member Koit Kõrgnurm, carrying a codon-optimized dCas9 and a non-targeting sgRNA was used as the backbone for the construction of the C-plasmid. As a first step, dCas9 was changed to Cas9 by A10D and A840H substitutions using a modified Quick-change PCR protocol,^31^ resulting in plasmid pGFT138. Then, the *repH* origin of replication of pGFT138 was replaced with *repA* using overlap extension PCR^32^ followed by replacing the *mlsR* resistance gene (for CLA) with the *catP* resistance gene (for TMP) via restriction-ligation (RL)-based cloning to obtain plasmid pGFT158. Next, the Pthl promoter of Cas9 on pGFT158 was swapped using the RL-based cloning with the CAETHG_4038 promoter native to *C. authoethanogenum*,^25^ resulting in the N-plasmid (pGFT165). To construct the C-plasmid (pGFT167), the non-targeting sgRNA on the N-plasmid was replaced with a sgRNA targeting the ColE1 origin of replication (designed using the CRISPR RGEN Tool^33^) via the Quick-change PCR protocol.^11^ Tables S6 and S7 list the plasmids and primers, respectively, used or constructed in this study.

### Preparation of *C. autoethanogenum* ECCs and Electroporation

The preparation of ECCs and electroporation of *C. autoethanogenum* strains were performed as described previously.^10,34^ Briefly for ECCs, a glycerol stock was revived and subcultured twice on YTF, then transferred to YTF supplemented with 40 mM dl-threonine, harvested at optical density (OD) 0.2–0.3, and washed twice with SMP buffer (270 mM sucrose, 1 mM MgCl_2_, 7 mM sodium phosphate; pH 6), and ECC cell pellets were suspended in 200 μL of SMP buffer containing 10% DMSO and stored as 25 μL aliquots at −80 °C. All steps except for centrifugation were carried out in an anaerobic chamber while keeping cells ice-chilled. Briefly for electroporation, 25 μL of ECCs were mixed with 2 μg of plasmid, transferred to a prechilled 1 mm electroporation cuvette, and electroporated at 625 V, 600 Ω, and 25 μF using the Gene Pulser Xcell (BioRad).^34^ Immediately, 1 mL of YTF was added to the cuvette and transferred to a serum bottle with 10 mL of YTF. After 20–24 h of recovery at 37 °C with gentle agitation (120 rpm), cultures were plated on selective and non-selective YTF agar plates.

### Plasmid Curing by Transformative Electroporation of ECCs Using the C- or N-Plasmid (no. 1 and no. 2 on Figure 1B)

The C- and N-plasmids were electroporated in two or three replicates into deletion strain ECCs, while the C-plasmid was also electroporated into LAbrini ECCs. For TMP and TMP + CLA plates each, 4.5 mL of the ∼10 mL electroporation recovery culture was transferred into two 15 mL centrifugation tubes, centrifuged at 5000 *g* for 3 min at ambient temperature, and resuspended in 150 μL of YTF before plating. For CLA and NS plates, the remaining 1 mL recovery culture was diluted to OD ∼0.1, serially diluted 1000-fold and 10,000-fold from which 150 μL were plated. Plates were incubated in Oxoid AnaeroJars (Thermo Fisher Scientific) at 37 °C until colonies appeared. Plasmid curing efficiency was determined by screening 10–12 colonies from NS plates using selective and non-selective growth tests in 1.5 mL microcentrifuge tubes. First, colony outgrowth was achieved in 3–4 days in 700 μL NS YTF at 37 °C. Second, 20 μL of culture was subcultured into 700 μL NS YTF, TMP YTF, and CLA YTF and incubated for 3–5 days. Colony was determined to be cured of plasmids if growth occurred in NS YTF but not in either of the selective YTF cultures. DNA extracted from the outgrowth cultures was used for PCR and gel-electrophoresis for confirming *C. autoethanogenum*, respective gene deletions, and presence of plasmids.

### Plasmid Curing by Non-transformative Electroporation of ECCs (no. 3 on Figure 1B)

Deletion strain ECCs were electroporated without a plasmid, 1000-fold and 10,000-fold dilutions of the recovery cultures were plated on NS YTF and CLA YTF agar, and plasmid curing efficiency was determined as described for the transformative electroporation of ECCs approach above.

### Plasmid Curing by Making Cells Electrocompetent (no. 4 on Figure 1B)

Deletion strain ECCs were inoculated into YTF recovery medium, plated, and screened as described for the non-transformative electroporation of ECCs approach above.

### Plasmid Curing by Buffer Wash and Non-transformative Electroporation (no. 5 on Figure 1B)

Engineered *C. autoethanogenum* and NEBExpress *E. coli* glycerol stocks (non ECCs) were pelleted, washed twice with 1.5 mL of buffer (SMP for *C. autoethanogenum* and 10% glycerol for *E. coli*), and resuspended in 25 μL of buffer for electroporation without a plasmid. *E. coli* electroporation was carried out as described before,^35^ except 2 kV was used. Plating and screening were done as described for the non-transformative electroporation of ECCs approach above, except SOB medium (20 g/L Bacto Tryptone, 5 g/L Yeast extract, 8.6 mM NaCl, 250 mM KCL, 10 mM MgCl_2_, 10 mM MgSO_4_, pH 7.0) was used for *E. coli*.

## Supplementary Material



## Notes

Additional experimental details with lists of strains, plasmids, and primers; results of plate counts, screening for plasmid curing, statistical tests between curing approaches, and PCR plus gel electrophoresis for pCA plasmid detection; gel images and Sanger sequencing data from C-plasmid construction steps; and plasmid maps (PDF)
