# Supplementary material for: Optimization of Plasmid Curing from Genetically Engineered Clostridium autoethanogenum
Source: ACS Synth Biol. 2025 Dec 2;14(12):4967–72. doi: 10.1021/acssynbio.5c00456 (PMC12723745; doi:10.1021/acssynbio.5c00456)
Supplement: Supplementary file 1 [file sb5c00456_si_001.pdf]

Supporting Information

Optimization of plasmid curing from genetically-engineered *Clostridium autoethanogenum*

Victoria Chinonyerem Udemezue[1], Kurshedaktar Majibullah Shaikh[1], Mariia Vorontsova[1], and Kaspar Valgepea[1,*]

[1]Institute of Bioengineering, University of Tartu, 50411 Tartu, Estonia

*Correspondence: Kaspar Valgepea, kaspar.valgepea@ut.ee

Supporting Table S1. Screening results of E-plasmid curing during serial non-selective sub-culturing of Δ*lpdA*. Cured colonies from liquid growth tests were determined by lack of growth in both of the selective media (see main text for details).

Sub-culture #	Colonies screened	Cured colonies (liquid growth test)	Plasmid Curing %
**3**	3	0	0
**4**	6	0	0
**5**	20	0	0
**6**	25	0	0
**7**	10	0	0
**8**	8	0	0

Supporting Table S2. Plate count results for various strains and curing approaches. All tested approaches for strains Δ*lpdA* and Δ*gcvH*Δ*acsV* and approach #5 for strains Δ*cooC2*, Δ*spo0A,* CLAU_1957 SNP*,* and *E. coli*. Additionally, results of transforming LAbrini with C- or N-plasmid. TMP, thiamphenicol selection plate (for C/N-plasmid); CLA, clarithromycin selection plate (for E-plasmid); Amp, ampicillin selection plate (for *E. coli*); NS, non-selective plate (without antibiotic); NP, not plated; NC, not counted.

Approach	Strain	Bio-replicate	TMP + CLA	TMP	CLA/Amp		NS	
					10^{-3}	10^{-4}	10^{-3}	10^{-4}
**C-plasmid electroporation of ECCs (#1)**	Δ*lpdA*	A	0	0	180	48	~1000	257
		B	0	1	254	42	~620	165
		C	0	0	304	29	434	102
	Δ*gcvH*Δ*acsV*	A	0	0	43	4	252	32
		B	24	0	260	26	1200	159
	LAbrini	A	NP	0	NP	NP	NP	NP
		B	NP	0	NP	NP	NP	NP
		C	NP	0	NP	NP	NP	NP
**N-plasmid electroporation of ECCs (#2)**	Δ*lpdA*	A	6	1	130	12	~1000	139
		B	16	9	295	32	212	135
		C	7	12	148	21	268	85
	Δ*gcvH*Δ*acsV*	A	0	0	61	13	300	44
		B	0	0	65	7	352	52
	LAbrini	A	NP	124	NP	NP	NP	NP
		B	NP	309	NP	NP	NP	NP
		C	NP	396	NP	NP	NP	NP
**Non-transformative electroporation of ECCs (#3)**	Δ*lpdA*	A	NP	NP	57	6	175	34
		B	NP	NP	36	6	215	33
		C	NP	NP	190	12	388	59
	Δ*gcvH*Δ*acsV*	A	NP	NP	208	19	286	62
		B	NP	NP	129	22	416	43
**ECC preparation (#4)**	Δ*lpdA*	A	NP	NP	23	2	187	64
		B	NP	NP	135	11	262	18
		C	NP	NP	67	10	312	30
	Δ*gcvH*Δ*acsV*	A	NP	NP	140	14	735	136
		B	NP	NP	144	16	496	86
**Non-transformative electroporation of buffer-washed glycerol stocks (#5)**	Δ*lpdA*	A	NP	NP	188	24	304	31
		B	NP	NP	15	1	66	5
		C	NP	NP	92	20	128	8
	Δ*gcvH*Δ*acsV*	A	NP	NP	NC	NC	85	13
		B	NP	NP	NC	NC	14	2
		C	NP	NP	NC	NC	211	18
	Δ*cooC2*	A	NP	NP	540	100	764	102
		B	NP	NP	405	122	823	126
		C	NP	NP	85	5	149	9
		D	NP	NP	147	12	169	23

	Δ*spo0A*	A	NP	NP	3	0	~1000	120
		B	NP	NP	0	0	~804	163
		C	NP	NP	0	0	~1000	123
	CLAU_1957 SNP	A	NP	NP	0	0	~1000	136
		B	NP	NP	0	0	~820	117
		C	NP	NP	0	0	~692	96
	E. coli NEBExpress	A	NP	NP	189	33	~5000	~812
		B	NP	NP	282	31	~5760	~1192
		C	NP	NP	300	57	~5168	364

Supporting Table S3. PCR and gel screening results of plasmid curing for all tested approaches for strains Δ*lpdA* and Δ*gcvH*Δ*acsV*, and of approach #5 for strains Δ*cooC2,* Δ*spo0A,* CLAU_1957 SNP*, E. coli*. Cured colonies from liquid growth tests were determined by lack of growth in both of the selective media (see main text for details). NA, not applicable.

Approach	Strain	Bio-replicate	Colonies screened	Colonies without plasmid band on gel after PCR		Colonies without bands for either plasmid	Plasmid Curing %	AVG plasmid curing %
				C/N-plasmid	E-plasmid			
**C-plasmid electroporation of ECCs (#1)**	Δ*lpdA*	A	10	10	4	4	40	56
		B	10	10	5	5	50	
		C	12	12	9	9	75	
	Δ*gcvH*Δ*acsV*	A	10	5	6	4	40	20
		B	10	2	3	0	0	
**N-plasmid electroporation of ECCs (#2)**	Δ*lpdA*	A	10	10	5	5	50	65
		B	12	12	10	10	83.3	
		C	12	12	7	7	58.3	
	Δ*gcvH*Δ*acsV*	A	10	5	9	5	50	60
		B	10	8	9	7	70	
**Non-transformative electroporation of ECCs (#3)**	Δ*lpdA*	A	10	NA	0	0	0	0
		B	10	NA	0	0	0	
		C	12	NA	0	0	0	
	Δ*gcvH*Δ*acsV*	A	10	NA	6	6	60	55
		B	10	NA	5	5	50	
**ECC preparation (#4)**	Δ*lpdA*	A	10	NA	0	0	0	10
		B	10	NA	1	1	10	
		C	10	NA	2	2	20	
	Δ*gcvH*Δ*acsV*	A	10	NA	7	7	70	75
		B	10	NA	8	8	80	
**Non-transformative electroporation of buffer-washed glycerol stocks (#5)**	Δ*lpdA*	A	10	NA	1	1	10	27
		B	10	NA	7	7	70	
		C	10	NA	0	0	0	
	Δ*gcvH*Δ*acsV*	A	10	NA	3	3	30	13
		B	10	NA	1	1	10	
		C	10	NA	0	0	0	
	Δ*cooC2*	A	12	NA	1	1	8.3	6.4
		B	12	NA	1	1	8.3	
		C	12	NA	0	0	0	
		D	11	NA	1	1	9.1	
	Δ*spo0A*	A	10	NA	10	10	100	100
		B	10	NA	10	10	100	
		C	10	NA	10	10	100	
	CLAU_1957 SNP	A	10	NA	10	10	100	100
		B	10	NA	10	10	100	
		C	10	NA	10	10	100	

	E. coli NEBExpress	A	10	NA	10	10	100	97
		B	10	NA	10	10	100	
		C	10	NA	9	9	90	

Supporting Table S4. Student's T-tests for statistical significance between plasmid curing efficiencies of approaches #2–5 compared to #1 (C-plasmid approach) for Δ*lpdA* and Δ*gcvH*Δ*acsV*. Data for tests from Figure 2 in main text.

Strain	Plasmid curing approach	#2	#3	#4	#5
ΔlpdA	**#1**	0.12	0.93	1.00	0.01
ΔgcvHΔacsV	**#1**	0.11	0.10	0.42	0.04

Supporting Table S5. List of strains used in this study.

Strain	Description	Genotype	Source
**NEB Turbo *E. coli***	Cloning and plasmid propagation	F' proA*B* lacI^q ΔlacZM15 / fhuA2 Δ(lac- proAB) glnV galK16 galE15 R(zgb210::Tn10)Tet^S endA1 thi-1 Δ(hsdS-mcrB)5	NEB
**Competent dcm- NEBExpress *E. coli***	No DNA methylation activity at DCM site (CCWGG).	fhuA2 [lon] ompT gal sulA11 R(mcr-73::miniTn10--TetS)2 [dcm] R(zgb-210::Tn10--TetS) endA1 Δ(mcrC-mrr)114::IS10	NEB
***C. autoethanogenum* LAbrini**	Isolate from adaptive laboratory evolution of *C. autoethanogenum*	Genbank CP110420	(Ingelman et al., 2024)
C. autoethanogenum* Δ*lpdA	LAbrini with deletion of *lpdA* gene possessing the editing plasmid	LAbrini Δ*lpdA*	From fellow group member U.J. Nwaokorie
C. autoethanogenum* Δ*gcvH*Δ*acsV	LAbrini with deletion of *gcvH* and *acsV* genes possessing the editing plasmid	LAbrini Δ*gcvH*Δ*acsV*	From M. Vorontsova
C. autoethanogenum* Δ*cooC2	LAbrini with deletion of *cooC2* gene possessing the editing plasmid	LAbrini Δ*cooC2*	From fellow group member U.J. Nwaokorie
C. autoethanogenum* Δ*spo0A	JA1-1 with CLAU_3129 (*spo0A*) deletion possessing the editing plasmid	JA1-1 Δ*spo0A*	(Ingelman et al., 2024)
***C. autoethanogenum* CLAU_1957 SNP**	JA1-1 with a SNP in CLAU_1957 gene possessing the editing plasmid	JA1-1 CLAU_1957 SNP	(Ingelman et al., 2024)

Supporting Table S6. Plasmids used or constructed in this study.

Plasmid Name	Description and Relevant Genetic Part	Source
**pGFT096**	Used as a template for the construction of C-plasmid. Genetic parts: Pthl-SpdCas9 (A10D, A840H), P_{J23119}, *repH, mlsR*; non-targeting gRNA	From fellow group member K. Kõrgnurm
**pGFT138a**	Substitution of alanine to aspartic acid at position 10 of dCas9 of pGFT096. Genetic parts: Pthl-SpCas9, P_{J23119}, *repH, mlsR*, non-targeting gRNA	This study
**pGFT138**	Substitution of alanine to histidine at position 840 of dCas9 of pGFT138a. Genetic parts: Pthl-SpCas9, P_{J23119}, *repH, mlsR,* non-targeting gRNA	This study
**pGFT149**	*repH* of pGFT138 replaced with *repA*. Genetic parts: Pthl-SpCas9, P_{J23119}, *repA*, *mlsR,* non-targeting gRNA	This study
**pGFT158**	*mlsR* of pGFT149 replaced with *catP*. Genetic parts: Pthl-SpCas9, P_{J23119}, *repA, catP*, non-targeting gRNA	This study
**pGFT165 (N-plasmid)**	Pthl promoter of pGFT158 replaced with CAETHG_4038 promoter. Genetic parts: CAETHG_4038-SpCas9, P_{J23119}, *repA, catP*, non-targeting gRNA	This study
**pGFT167 (C-plasmid)**	gRNA of N-plasmid (pGFT165) swapped with ColE1_gRNA3. Genetic parts: CAETHG_4038-SpCas9, P_{J23119}, ColE1_gRNA3, *repA, catP*	This study
**pGFT123**	Plasmid used for amplifying the CAETHG_4038 promoter	From fellow group member A. Pagliotto
**pGFT107**	Plasmid used to transform *E. coli* NEBExpress for testing plasmid curing from *E. coli* with approach #5.<br>Genetic parts: Pthl-SpnCas9, P_{J23119}, *repH, mlsR*; spo0A gRNA	(Ingelman et al., 2025)

Supporting Table S7. Primers used in this work. The bold black sequence is the point of codon mutation, the bold green sequence is the overlapping region with the template plasmid for *repA* amplification, and the bold blue sequence is the gRNA spacer sequence.

Primer Name	Primer sequence	Usage
Cas9_FP	tagtattggtctt**gat**ataggaacaaatagtgtaggatg	Cas9 A10D mutation
CA_Cas9_mut10_RP	tatttgttcctat**atc**aagaccaatactatattttttatccatcc	Cas9 A10D mutation
CA_Cas9_mut840_FP	atgatgtagat**cat**atagttcctcaaagttttcttaaagatgattc	Cas9 A840H mutation
CA_Cas9_mut840_RP	gaggaactat**atg**atctacatcataatctgaaagtctattaatatcaag	Cas9 A840H mutation
pBP1_FP_OE	ctgcatcccttaacttgtttttcgtgtacctattttttgtgaatcgat**ggcgcgccgttctgaatcc**	*repA* amplification
pBP1_RP_OE	cctaggactgagctagctgtcaactgcagcacattaag**cttcggccggccgaatttgtaaataaacc**	*repA* amplification
catP_SacI_FP	aattatgagctcggccggccagtgggcaag	*catP* amplification
catP_ClaI_RP	aattatatcgatgttaactatttatcaattcctgcaattcgtttacaaaacggcaaatgtg	*catP* amplification
repA_Seq_RP	catcttccgtaagttcccttattctag	For sequencing and C/N-plasmid verification
catP_Seq_FP	gcaacggtatggaaacaatcatagaatgg	For sequencing and C/N-plasmid verification
ColE1_gRNA1	gtcctaggtataatactagt**cagaggtggcgaaacccgac**gttttagagctagaaatagcaagttaaa	gRNA swapping
ColE1_gRNA2	gtcctaggtataatactagt**agttaccggataaggcgcag**gttttagagctagaaatagcaagttaaa	gRNA swapping
ColE1_gRNA3	gtcctaggtataatactagt**gtaacaggattagcagagcg**gttttagagctagaaatagcaagttaaa	gRNA swapping
pNC_gRNA_OE_R	actagtattatacctaggactgagctagctgtcaactgcagc	gRNA swapping
CAETHG_4038-150-XhoI-F	atatctcgagtgtggaatgaggaaaaaaatattag	CAETHG_4038 promoter amplification
SmaI_CAETHG_4038_150_RP	atatatcccgggaatagtaacctccattcttattcctttatgg	CAETHG_4038 promoter amplification
pIPL12-Cas9-ctrl-R	gttatttgtctagtttctacaagctgtc	For sequencing
pIPL12_Cas9_seq_RP	agcttctgctgtttcacctgaatc	For sequencing and C/N plasmid verification
NickClos-1-F	gtcgttgtttaggtgtgttgtaatac	E-plasmid and pGFT107 verification
SmaI-Pthl-R	atccatcccgggtctaactaa	E-plasmid and pGFT107 verification

acsB-F	ggtgcattagatatagtaagaagtttg	*acsB* gene amplification for *C. autoethanogenum* verification
acsB-R	acttctccctgatctgcacac	*acsB* gene amplification for *C. autoethanogenum* verification
lpdA_KO-F	ggtgaagtagaaagagttggaca	Δ*lpdA* verification
lpdA_KO-R	ggtatttcctgtatctccatcaa	Δ*lpdA* verification
catP_short_F	gtgtaccttgtacctacagcatg	C/N-plasmid verification
catP_short_R	gtcacatactgcgtgatgaacttg	C/N-plasmid verification
mlsR_short_F	gtacagaggtgtaatttcgtaactgc	E-plasmid verification
mlsR_short_R	gttaagggatgcagtttatgcatc	E-plasmid verification
pCA-F	gttatttgaaacagcgtcatttaaataaaaaatatctgg	pCA verification
pCA-R	cgaaatttaagtgcatatgtggagtggattc	pCA verification
PCA-F2	ctgataaaacccaaaaaggtgaagaaagacc	pCA verification
PCA-R2	aaccttcttcacattctttaacattacaactcac	pCA verification

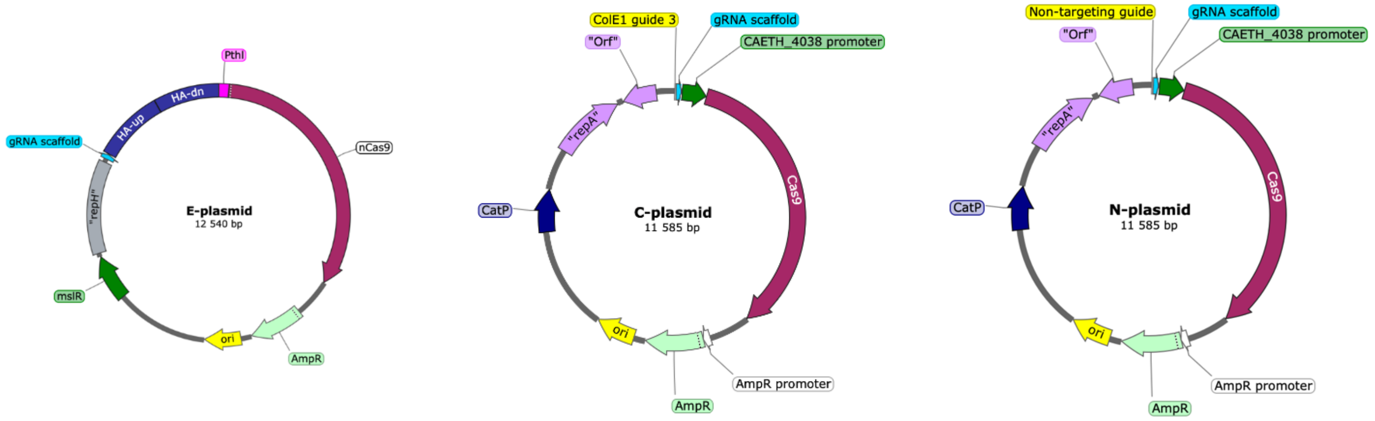

Supporting Figure S1. Plasmids maps for *C. autoethanogenum* editing (E-plasmid), curing (C-plasmid) and control plasmids (N-plasmid) constructed in this study.

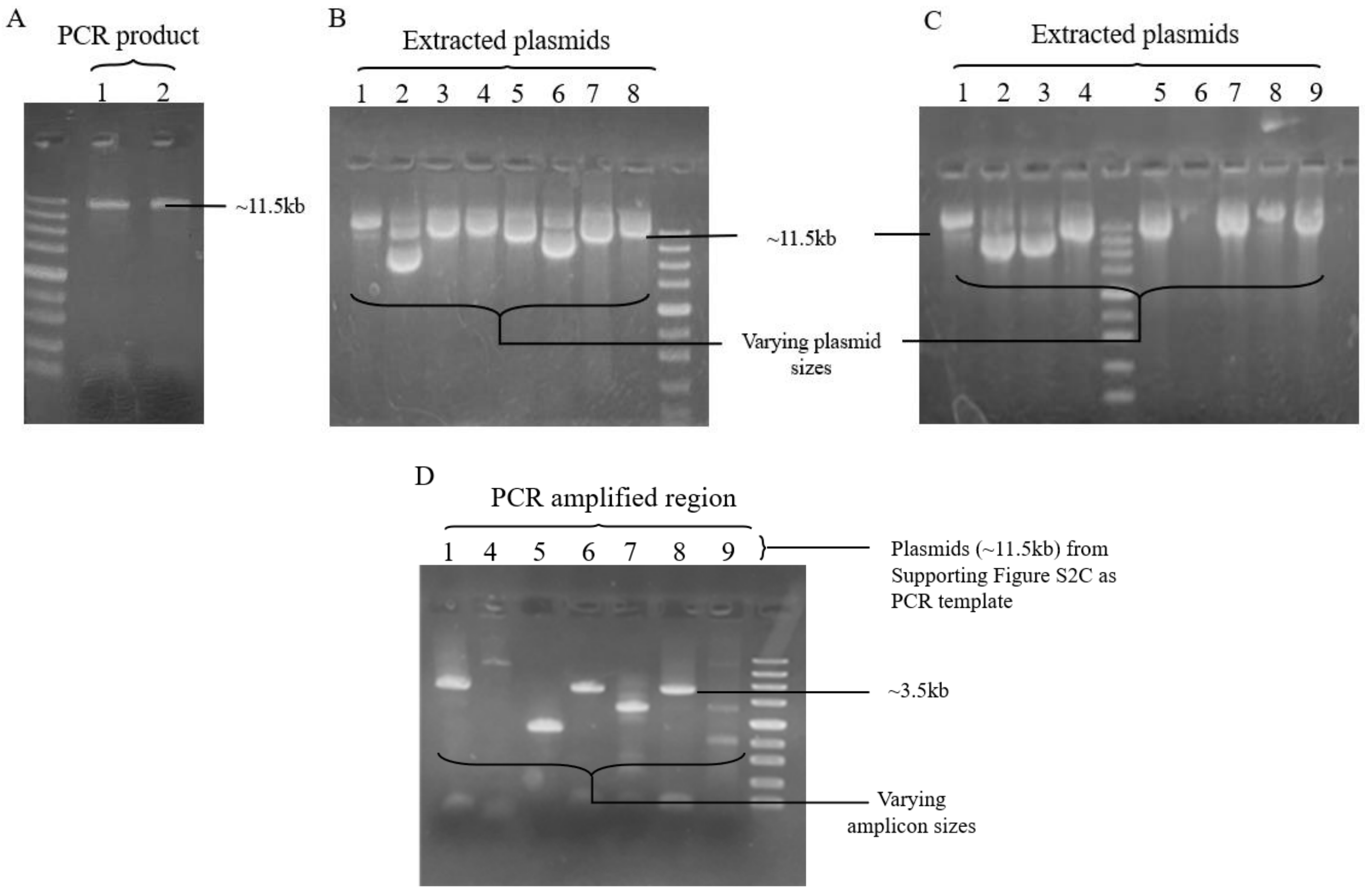

Supporting Figure S2. Gel images from C-plasmid construction steps. (A) PCR product from overlap-extension PCR for swapping the non-targeting gRNA with ColE1_gRNA3. (B) and (C) Extracted plasmid from transformants observed from gRNA swapping (undigested). (D) PCR amplified region of ~3.5 kb using extracted plasmids from panel (B) and (C) as template.

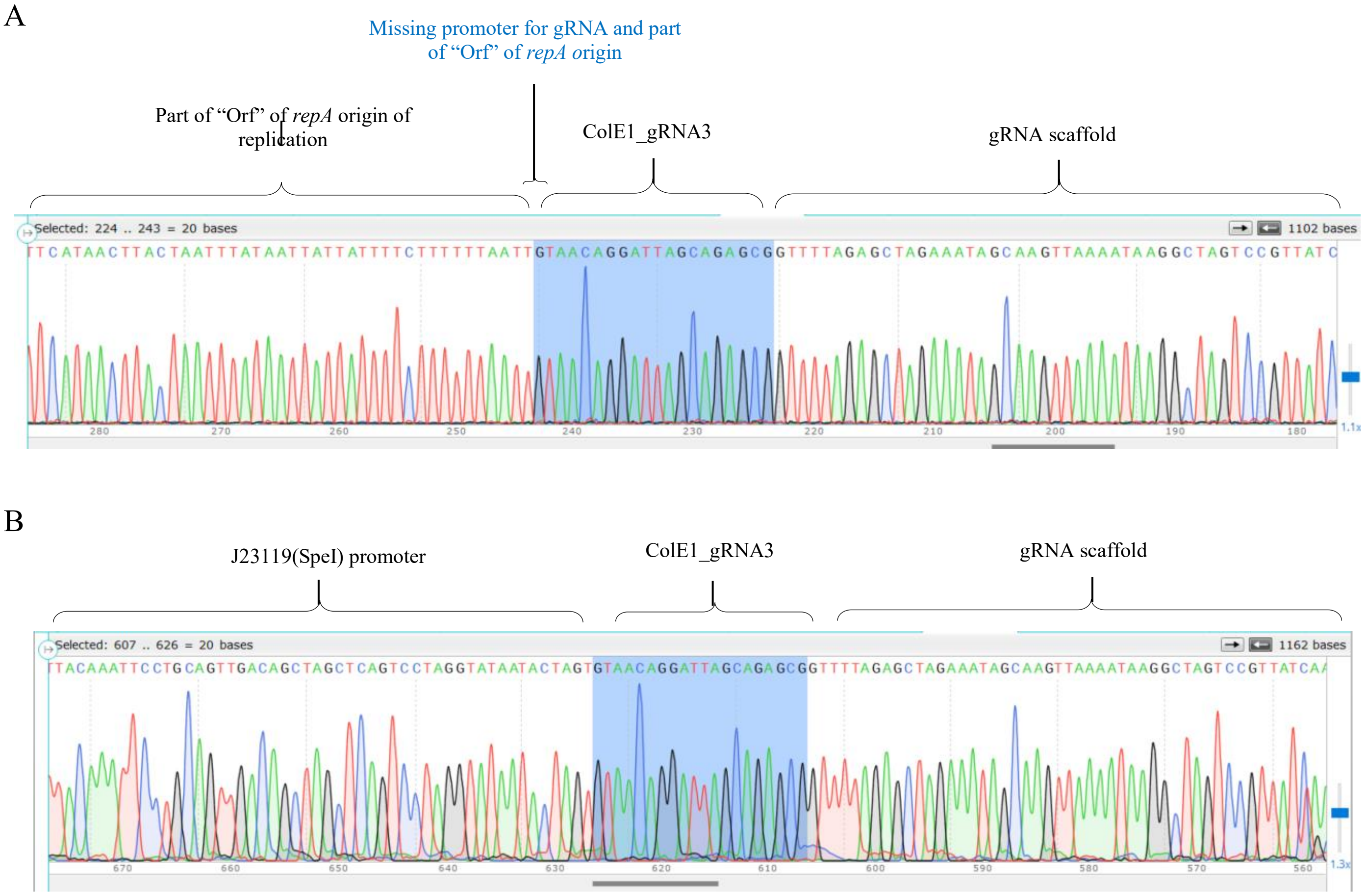

Supporting Figure S3. Sanger sequencing results of the amplified gRNA expression cassette region for the incorrect plasmid with missing genetic parts and the C-plasmid. (A) Plasmid with missing genetic parts: J23119 (SpeI) promoter and part of "Orf" of *repA* origin of replication. (B) Correct gRNA spacer sequence targeting Col_E1 origin of replication made using Col_E1_gRNA3 and complete gRNA expression cassette.

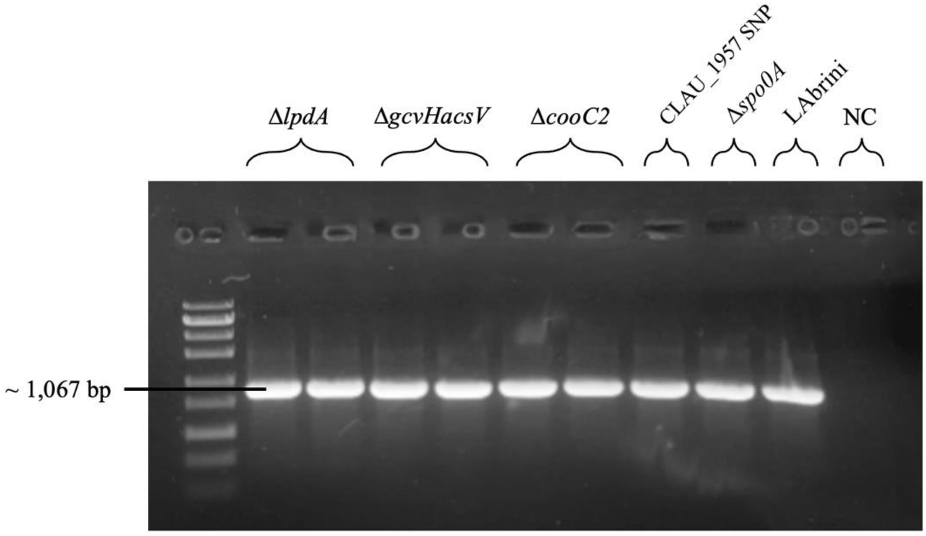

Supporting Figure S4. Gel image for the PCR-amplified region from the native plasmid pCA in *C. autoethanogenum* LAbrini, uncured genetically-engineered JA1–1 strains (CLAU-1957 SNP and Δ*spo0A*), and genetically-engineered LAbrini strains (Δ*lpdA,* Δ*cooC2,* and Δ*gcvH*Δ*acsV)* cured of E-plasmids. JA1–1 strains were used as controls. The expected PCR product size is ~1kb.
